# Glymphatic System as a Gateway to Connect Neurodegeneration From Periphery to CNS

**DOI:** 10.3389/fnins.2021.639140

**Published:** 2021-02-09

**Authors:** Gianfranco Natale, Fiona Limanaqi, Carla L. Busceti, Federica Mastroiacovo, Ferdinando Nicoletti, Stefano Puglisi-Allegra, Francesco Fornai

**Affiliations:** ^1^Department of Translational Research and New Technologies in Medicine and Surgery, University of Pisa, Pisa, Italy; ^2^IRCCS Neuromed, Pozzilli, Italy

**Keywords:** glymphatic system, lymphatic system, blood–brain barrier, neurovascular unit, neurodegenerative diseases

## Abstract

The classic concept of the absence of lymphatic vessels in the central nervous system (CNS), suggesting the immune privilege of the brain in spite of its high metabolic rate, was predominant until recent times. On the other hand, this idea left questioned how cerebral interstitial fluid is cleared of waste products. It was generally thought that clearance depends on cerebrospinal fluid (CSF). Not long ago, an anatomically and functionally discrete paravascular space was revised to provide a pathway for the clearance of molecules drained within the interstitial space. According to this model, CSF enters the brain parenchyma along arterial paravascular spaces. Once mixed with interstitial fluid and solutes in a process mediated by aquaporin-4, CSF exits through the extracellular space along venous paravascular spaces, thus being removed from the brain. This process includes the participation of perivascular glial cells due to a sieving effect of their end-feet. Such draining space resembles the peripheral lymphatic system, therefore, the term “glymphatic” (glial-lymphatic) pathway has been coined. Specific studies focused on the potential role of the glymphatic pathway in healthy and pathological conditions, including neurodegenerative diseases. This mainly concerns Alzheimer’s disease (AD), as well as hemorrhagic and ischemic neurovascular disorders; other acute degenerative processes, such as normal pressure hydrocephalus or traumatic brain injury are involved as well. Novel morphological and functional investigations also suggested alternative models to drain molecules through perivascular pathways, which enriched our insight of homeostatic processes within neural microenvironment. Under the light of these considerations, the present article aims to discuss recent findings and concepts on nervous lymphatic drainage and blood–brain barrier (BBB) in an attempt to understand how peripheral pathological conditions may be detrimental to the CNS, paving the way to neurodegeneration.

## Introduction: Classic Anatomical Concepts

Apart from the general protection provided by the skull and dura mater, the brain environment is rigidly regulated by specialized structures, including leptomeninges, modified blood vessels and glial cells. In particular, selective capillaries, astrocyte end-feet, and pericytes represent the classic components of the blood–brain barrier (BBB). This barrier provides the brain with nutrients, transports catabolites, and misfold-prone proteins out of the brain, maintains brain homeostasis, and regulates the immune function (Ballabh et al., 2004; Daneman and Prat, 2015; Hladky and Barrand, 2018).

This anatomical barrier, as postulated by Stern and Gautier (1921), regulates the molecular exchange between the blood flow and brain parenchyma, thereby controlling homeostasis within central nervous system (CNS). Apart from being a route of drainage for brain interstitial fluid (ISF) to lymph nodes, these structures provide the communication with the immune system modulating surveillance and immune-mediated responses to the brain. However, similar barriers also deputed to the regulation of molecular transport and immunologic protection are described outside the CNS. This is the case of the retina (part of the blood–ocular barrier), placenta, testis (seminiferous tubules), and thymus cortex. These barriers possess a well-defined anatomical substrate, since both endothelium and epithelial cells adjacent to capillaries exhibit special intercellular junctions (Fröhlich, 2002).

**FIGURE 1:**
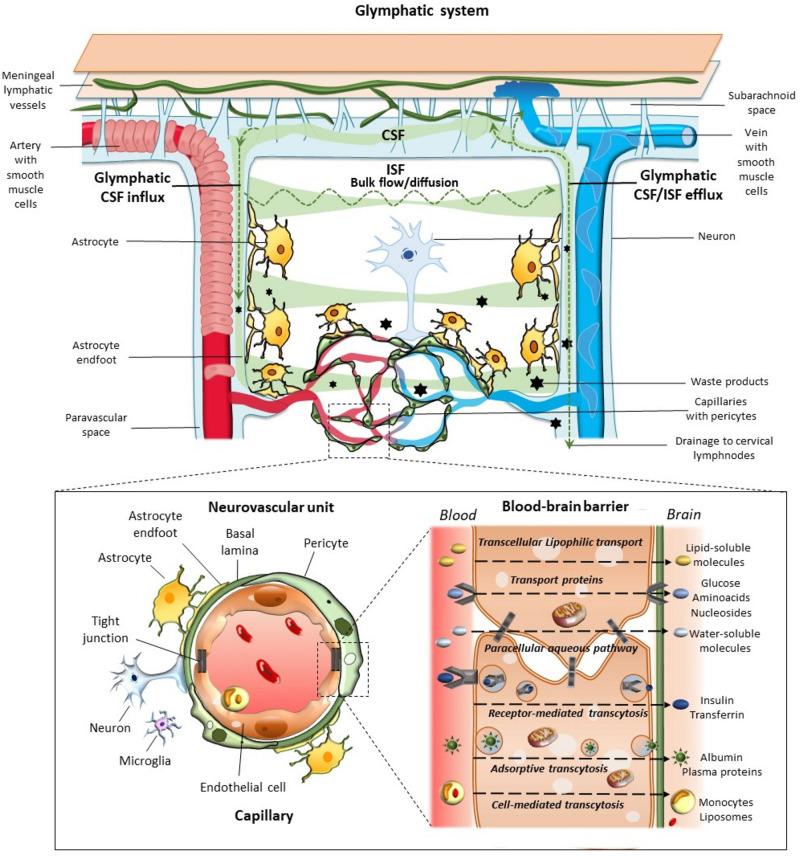
Glymphatic system, neurovascular unit (NVU), and the blood–brain-barrier. The glymphatic system contributes to the transport of nutrients and signaling molecules into the brain parenchyma meanwhile promoting the clearance of proteins and interstitial waste solutes out of the brain. Subarachnoid CSF enters the brain parenchyma via para-arterial spaces and then mixes with the interstitial fluid (ISF) and waste solutes in the parenchyma. Whether this occurs through convective bulk flow or diffusion remains debated. The resulting CSF-ISF fluid exchange and the interstitial waste solutes enter the paravenous space through gaps between the astrocytic end-feet to be drained either back to the CSF-dural sinus-meningeal lymphatic vessels, or to the deep cervical lymph nodes. Green arrows and shades indicate the CSF and CSF-ISF fluid transport, while black stars indicate the interstitial waste solutes that exit the parenchyma via the paravenous efflux pathway. The insert depicts the main components of the NVU at the level of intraparenchymal capillaries, including perivascular astrocytes with their end-feet, neurons, microglia, pericytes, endothelial cells (ECs), and basement membrane (basal lamina). Capillary ECs are held together by tight junctions forming the blood–brain barrier (BBB), where the different transport routes are represented, including transcellular lipophilic transport, carrier protein-mediated transport, paracellular aqueous transport, receptor-mediated transcytosis, as well as adsorptive and cell-mediated transcytosis.

Morphological and functional findings allowed to look at the BBB from novel perspectives. For instance, the specialized metabolic interface of BBB can also act as a target for hormones and may secrete active compounds (Banks, 2019). The intimate relationship between CNS and blood vessels was deeply modified, when at the 2001 Stroke Progress Review Group Meeting of the National Institute of Neurological Disorders and Stroke the concept of the neurovascular unit (NVU) was formalized (Iadecola, 2017). Its cellular components include endothelial cells (ECs), basement membrane, (BM), perivascular astrocytes, neurons, pericytes, and microglia (Figure 1). As suggested by its name, this minimal functional unit emphasizes the relationship between CNS and blood vessels. In fact, a focally specific activity of a given NVU may alter locally the anatomy and physiology of the BBB, apart from controlling the amount of cerebral blood flow within the same specific region. ECs represent the major BBB component, being endowed with tight and adherent junctions between adjacent cells, which prevent paracellular diffusion of polar blood solutes while providing structural support (Daneman and Prat, 2015; Giorgi et al., 2020). Proteins such as occludins, claudins, and cadherins are expressed in these junctions. Endothelial transporters ensure mechanisms for both influx and efflux of either potentially beneficial or harmful substances. Surrounding the epithelium, the BM provides anchoring support to blood vessels and surrounding cells with its extracellular matrix rich in collagen and proteoglycan (Bell et al., 2020; Giorgi et al., 2020). Astrocytes lie between neurons and ECs, and with their end-feet surround blood vessels at precapillary and capillary level. Thus, astrocytes provide structural and functional connection between blood vessels and neurons. Neurons are particularly sensitive to changes of blood oxygen and nutrients, then acting as metabolic pacemakers. Apart from ion and neurotransmitter recycling, astrocytes are involved in BBB induction and maintenance through the release of several growth factors, regulation of dilation and constriction of blood vessels, as well as water balance within the interstitial space through the expression of aquaporin-4 (AQP4) at the level of their end-feet (Daneman and Prat, 2015; Giorgi et al., 2020). Pericytes also participate in BBB development, structural integrity, and function through the production and assembling of BM proteins, as well as regulation of tight junction expression, and EC proliferation (Armulik et al., 2010; Giorgi et al., 2020). Thanks to the presence of contractile proteins, pericytes have also been involved in blood flow regulation (Yamazaki and Kanekiyo, 2017; Bell et al., 2020; Giorgi et al., 2020). Finally, microglia and phagocytes in the extracellular matrix surrounding blood vessels play a waste-clearing and immunological role (Giorgi et al., 2020).

The belief of an absence of conventional lymphatic vessels in the CNS contributed to the concept that the brain, in spite of its high metabolic rate, represents an immune privileged region. This idea left questioned how cerebral interstitial fluid is cleared from waste products. It was generally thought that clearance depended on cerebrospinal fluid (CSF), acting as a pseudo-lymphatic system. CSF is generally formed by choroid plexuses, which are protrusions located in cerebral ventricles consisting of a single layer of secretory epithelial cells (modified ependymal cells) that surround a core of capillaries and connective tissue. While epithelial cells are provided with tight junctions, capillaries are fenestrated. Then, within the general concept of BBB, choroid plexuses are a highly vascularized tissue that represents a different functional interface between blood and ventricular as well as subarachnoid spaces, constituting the so-called blood-CSF (or liquor) barrier (Kratzer et al., 2020). Overcoming these classic anatomical concepts, outer brain barriers are indeed composed of at least 3 interfaces, blood-CSF barrier across arachnoid barrier cell layer, blood-CSF barrier across pial microvessels, and outer CSF-brain barrier comprising glial end-feet layer/pial surface layer (Brøchner et al., 2015). Again, both pioneering and recent evidence has been provided pointing at extra-choroidal CSF production as well as novel mechanisms for CSF clearance (Sato and Bering, 1967; Milhorat, 1969; Milhorat et al., 1971; Orešković et al., 2017; Fame and Lehtinen, 2020). The ongoing CSF production and solutes transport from the blood to the CSF in choroid plexectomized rhesus monkeys suggests that the choroid plexus is probably not the sole or even the major source of CSF within the primate ventricular system (Sato and Bering, 1967; Milhorat, 1969; Milhorat et al., 1971). Accordingly, CSF production and absorption are constant and present everywhere in the CSF system, and the CSF is mainly formed as a consequence of water filtration between the capillaries and interstitial fluid (Orešković et al., 2017).

Highly permeable capillaries are present in specific brain regions, where a typical BBB is lacking and molecules freely diffuse from the blood into the brain. Since these areas are mostly placed between neural tissue and ventricle lumen, they are known as “circumventricular organs.” At this level, specialized ependymal cells, named tanycytes, equipped with a differential distribution of tight junction proteins, form a particular blood-CSF barrier. These circumventricular organs represent specialized neuro-epithelial regions, which include sensory (subfornical organ, area postrema, vascular organ of lamina terminalis) and secretory structures (median eminence, pituitary neural lobe, pineal gland). Then, these organs are important sites for communication with the CSF, as well as between brain and periphery by means of a rapid neuro-humoral exchange (Kaur and Ling, 2017).

The idea of a diffuse clearing process was replaced by the identification of anatomically and functionally discrete spaces surrounding the blood vessels of the brain (Bacyinski et al., 2017). These include the perivascular and paravascular spaces, where solute transport occurs in opposite directions. In detail, according to the perivascular model, ISF and solutes from the brain parenchyma enter the peri-arterial space in the BM of capillaries and within the tunica media of penetrating arteries (Carare et al., 2008). From the peri-arterial space, solutes (soluble antigens but not cells) are cleared from the brain by dispersing in CSF or draining directly into cervical lymph nodes (Weller, 2005; Carare et al., 2008; Bacyinski et al., 2017). A failure of such a perivascular drainage is associated with β-amyloid accumulation (Carare et al., 2008; Bakker et al., 2016). Solute clearance from the brain parenchyma to the cervical lymphatic system through the perivascular pathway occurs in a direction which is opposite to that of both blood flow and paravascular pathway (Carare et al., 2008; Weller et al., 2009; Abbott, 2013; Bakker et al., 2016; Bacyinski et al., 2017).

The paravascular space (Virchow-Robin or Durant-Fardel space of the classic literature) was described in terms of a pathway for the clearance of interstitial molecules (Iliff et al., 2012). This was documented through *in vivo* two-photon microscopy and *ex vivo* confocal microscopy in mice, and consists of a three-step pathway: (1) CSF enters the brain parenchyma along with arterial paravascular (extramural) spaces; (2) CSF is mixed with ISF and solutes in a process mediated by AQP4; and (3) CSF exits through the extracellular space (“transparenchymal” convection) along venous paravascular spaces to be removed from the brain. Since this process includes the participation of perivascular glial cells with a sieving effect of their end-feet, and it resembles the classic peripheral lymphatic system, the group of Maiken Nedergaard coined the term of “glymphatic” (glial-lymphatic) pathway (Iliff et al., 2012). Thus, the glymphatic system consists of a unidirectional fluid current flowing from the paravascular space of penetrating arteries and arterioles to that of large caliber parenchymal draining veins (Figure 1).

As far as the classic lymphatic system is concerned, studies of the past decade provided the first morphological, phenotypical and functional characterization of lymphatic vessels in the cerebral dura mater draining to the cervical lymph nodes (Aspelund et al., 2015; Louveau et al., 2015; Da Mesquita et al., 2018). It is suggested that meningeal lymphatic vessels absorb CSF from the adjacent subarachnoid space and brain ISF via the glymphatic system, thus acting as a drainage route for CSF while contributing to immune surveillance of the CNS (Aspelund et al., 2015; Louveau et al., 2015; Raper et al., 2016; Da Mesquita et al., 2018; Tamura et al., 2020). These studies also allowed the work of Paolo Mascagni, who was the first to describe in the 1787 a potential lymphatic system in the dura of humans in his masterpiece “Vasorum lymphaticorum corporis humani historia et ichnographia,” to be recognized and accepted by the scientific community (Natale et al., 2017; Irschick et al., 2019). This was reported by a number of additional works (Lukić et al., 2003; Bucchieri et al., 2015; Kumar et al., 2019; Sandrone et al., 2019; Mestre et al., 2020; Tamura et al., 2020). The presence of lymphatic vessels was demonstrated in the dura of both humans and non-human primates (Louveau et al., 2015; Absinta et al., 2017; Visanji et al., 2018). In humans, these vessels were detected at the level of the superior sagittal sinus and falx cerebri through immune-staining for podoplanin (a marker specific for lymphatic vessel ECs, Absinta et al., 2017; Visanji et al., 2018).

Thus, waste solutes may be ultimately cleared from the brain by draining into different compartments, including CSF-filled subarachnoid space and arachnoid villi, conduits along cranial and peripheral nerves, paravascular routes, as well as meningeal and cervical lymphatics (Iliff et al., 2012; Bedussi et al., 2015; Tarasoff-Conway et al., 2015; Raper et al., 2016; Benveniste et al., 2017; Ma et al., 2017). Similar to the perivascular clearance which requires cardiac output (Carare et al., 2008), cerebral arterial pulsation plays a pivotal role in driving glymphatic CSF influx into and through the brain parenchyma (Iliff et al., 2012). Thus, changes in arterial pulsatility may contribute to the accumulation of toxic solutes, including β-amyloid, in the aging brain (Iliff et al., 2013). Notwithstanding the importance of this discovery, characterization of such a highly organized CSF-ISF exchange pathway dates back to studies of the mid 80s by Patricia Grady’s group (Rennels et al., 1985). In fact, early evidence for a paravascular fluid circulation in the mammalian CNS was provided by the rapid and widespread distribution of a CSF tracer (horseradish peroxidase protein) throughout the brain from the subarachnoid space.

Nowadays, the glymphatic model has been further confirmed and highly praised (Jessen et al., 2015; Nistal and Mocco, 2018; Plog and Nedergaard, 2018; Sun et al., 2018; Benveniste et al., 2019; Kumar et al., 2019; Thomas et al., 2019; Mestre et al., 2020), and it was recently described in humans (Ringstad et al., 2018). The glymphatic pathway is being widely investigated comparing healthy and pathological conditions, such as neurodegenerative disorders, including chronic Alzheimer’s disease (AD), as well as hemorrhagic and ischemic stroke, hydrocephalus or traumatic brain injury. Descriptions in human disorders were backed up by experimental modeling of the system, which was developed to predict a potential site of therapeutic intervention (Rasmussen et al., 2018; Jiang, 2019; Ramos et al., 2019; Kaur et al., 2020; Reeves et al., 2020). Novel anatomical insights were provided indicating that the midbrain, due to the consistent thickness of its pial-glial BM, is better equipped for convective influx/glymphatic entry of the CSF compared with other brain regions. This may be a key for the intrathecal delivery of drugs into the brain (Dobson et al., 2017).

Nonetheless, the glymphatic model has been also revisited, and it represents a matter of debate. Many controversies and open issues exist concerning the perivascular and paravascular models, including the opposite directions of fluid flow, anatomical and functional differences, potential driving forces, and their role in health and disease (Bakker et al., 2016; Bacyinski et al., 2017). Some morphological and functional studies postulated an alternative hypothesis, which considers diffusion (not convective bulk flow) as the main mechanism regulating CSF-ISF exchange at the level of the NVU associated with brain capillaries, and throughout the interstitial space (Asgari et al., 2016; Jin et al., 2016; Smith et al., 2017; Abbott et al., 2018; Bakker et al., 2019; Kaur et al., 2020).

This is related to another major controversial aspect of the glymphatic hypothesis, which is centered on the role for AQP4 in CSF-ISF exchange under physiological conditions (Abbott et al., 2018). It has been argued that diffusion, rather than AQP4 expression, is an important regulator of paravascular flow, since CSF tracer uptake and interstitial flow rate are unaffected by ablation of the *Aqp4* gene (Smith et al., 2017). By using a cisternal infusion paradigm in mice similar to that employed by Iliff et al. (2012) and Smith et al. (2017) argued that tracer movement in the brain parenchyma outside of the perivascular spaces was size dependent and consistent with diffusion as the main mechanism of transport. Both studies differ on the anesthetics used, to which a subsequent study accessed the correlations of different anesthetics, electroencephalogram (EEG) power, and CSF tracer influx (Hablitz et al., 2019). Again, arguing against a major contribution of the ISF bulk flow model, several studies showed that most of β-amyloid removal occurs via the BBB (Deane et al., 2004, 2008; Tarasoff-Conway et al., 2015; Hladky and Barrand, 2018). Nonetheless, several studies confirmed that AQP4 inhibitors or *Aqp4* gene deletion slows down or impairs both glymphatic CSF tracer influx and the clearance of several interstitial solutes, including β-amyloid, ApoE, tau, SOD1 oligomers, lactate, and viruses (Iliff et al., 2014; Achariyar et al., 2016; Murlidharan et al., 2016; Lundgaard et al., 2017; Mestre et al., 2018; Feng et al., 2020; Harrison et al., 2020; Hirose et al., 2020).

Mice expressing normal AQP4 levels but specifically lacking perivascular AQP4 localization also exhibit impaired CSF tracer influx (Mestre et al., 2018). In this frame, pericytes play a key role in regulating AQP4 polarization in astrocytes end-feet (Gundersen et al., 2014). As support to a key role of pericytes in glymphatic function, pericyte-deficient *Pdgfb*^*ret/ret*^ mice feature both mispolarization of AQP4 from astrocyte end-feet, and defective glymphatic function (Armulik et al., 2010; Munk et al., 2019). In this same model, the development of the vasculature is more generally altered, including capillary dilation and impaired BBB function (Armulik et al., 2010). Several pieces of evidence now support a scenario in which pericytes influence the development of the glymphatic system through deposition of laminin 211 in the vascular BM, which via dystroglycan and dystrophin in astrocytes promotes polarization of AQP4 to its end-feet (Lendahl et al., 2019; Zheng et al., 2020).

Considering the vascular and metabolic importance of BBB and glymphatic system, alterations of these structures have been implicated in the pathogenesis of several neurological diseases. On the other hand, also in peripheral organs the human interstitial space has been revised and a novel concept of the space within and between cells has been proposed (Benias et al., 2018; Kumar et al., 2019). The present article aims to discuss recent findings in an attempt to envisage how perturbations of the glymphatic system can take a role in favoring or accelerating neurodegenerative processes within CNS. In particular, the involvement of peripheral alterations, central draining and clearing systems are considered in this intriguing relationship.

## Glymphatic System and CNS Disorders

The disruption of the brain (g)lymphatic system plays a crucial role in age-related changes of brain functions, as well as in the pathogenesis of neurovascular, neurodegenerative, neuro-inflammatory diseases, brain injury and tumors (Sun et al., 2018).

Several lines of evidence documented that β-amyloid and tau exit the brain via the glymphatic system, and glymphatic activity and CSF outflow decrease significantly in old mice (Iliff et al., 2014; Kress et al., 2014; Jessen et al., 2015; Ma et al., 2017). The glymphatic system removes potentially harmful metabolites from the CNS especially during sleep (Rasmussen et al., 2018; Hauglund et al., 2020). Accordingly, in animal models it was observed that during natural sleep or anaesthesia there is an enlargement of the interstitial space, which increases convective CSF exchange with ISF, and β-amyloid clearance rate (Xie et al., 2013). Again, obstructive sleep apnea increases cerebral β-amyloid aggregation and it is associated with increased prevalence of neurodegeneration, including AD (Ju et al., 2019). This is correlated with reduced slow wave activity (SWA). In fact, high SWA and certain types of anaesthesia support glymphatic activity, while norepinephrine signaling in the brain (and wakefulness, in general) has an attenuating effect (Xie et al., 2013; Hablitz et al., 2019; Hauglund et al., 2020). Disrupting the SWA is enough to abolish waste clearance (Ju et al., 2017), and sleep deprivation is correlated with increased levels of β-amyloid in the brain of both animals and humans (Kang et al., 2009; Shokri-Kojori et al., 2018). In line with this, the concentration of β-amyloid in CSF follows the sleep-wake cycle in AD human subjects, providing a correlation between bad sleep quality and β-amyloid deposition in the preclinical stage of AD (Ju et al., 2013).

In patients with AD, a decrease in both BBB and glymphatic function, accompanies a general dysfunction of NVU, including astrocytic end-feet atrophy, pericyte degeneration, alteration of endothelial tight junctions, and thickening of the basement membrane (Yamazaki and Kanekiyo, 2017). Consequently, CSF clearance of β-amyloid and tau tracers is reduced. This glymphatic dysfunction may be in part related to an altered AQP4 expression, as shown in different animal models of traumatic brain injury, AD, and stroke. In young APP/PS1 double transgenic mice, expressing chimeric mouse/human amyloid precursor protein (Mo/HuAPP695swe) and a mutant form of human presenilin-1 (PS1-dE9), it was shown a reduced glymphatic influx and clearance of β-amyloid, which worsens with aging. More in depth, glymphatic transport appeared suppressed in old APP/PS1mice, with β-amyloid deposits, and glymphatic clearance was reduced prior to the presence of β-amyloid deposits in younger APP/PS1 mice when compared to age-matched controls. As in a vicious circle, it was also shown that administration of wild-type mice with β-amyloid led to significant suppression of CSF tracer influx, suggesting that AD can cause a further reduction of glymphatic clearance (Peng et al., 2016). In fact, cerebral amyloid angiopathy consists of increased arterial stiffness, decreased arterial pulse, and reduction of perivascular spaces, due to extracellular β-amyloid accumulation (Peng et al., 2016; Plog and Nedergaard, 2018; Rasmussen et al., 2018; Reeves et al., 2020).

In the case of haemorrhagic stroke, the impairment of the glymphatic system is due to blood components, such as fibrin and fibrinogen deposits, which occlude perivascular spaces. In the ischaemic stroke there is an impaired CSF inflow and the release of several pro-inflammatory cytokines. Contrast-enhanced magnetic resonance imaging indicates that the glymphatic system is affected during stroke, although to a different extent, depending on the specific disorder [subarachnoid or intracerebral hemorrhage, carotid ligature, and embolic ischemic stroke (Gaberel et al., 2014)]. Moreover, cerebral drainage appeared affected also during multiple microinfarction, with inhibition of AQP4 function, as demonstrated in a murine model (Wang et al., 2017). Clearance of solutes, including tau protein, from the interstitial space is reduced by ∼60% after traumatic brain injury in experimental animals, with this impairment persisting for at least 1 month (Iliff et al., 2014).

An altered glymphatic function has been advocated to account for AD, as well as for idiopathic normal pressure hydrocephalus. The latter condition affects up to 10% of patients affected by dementia, who concomitantly suffer from idiopathic normal pressure hydrocephalus, with progressive ventriculomegaly, and the clinical triad of gait ataxia, urinary incontinence, and dementia (Reeves et al., 2020). In this respect, intrathecal contrast-enhanced magnetic resonance imaging has been suggested to diagnose pre-clinical neurodegenerative disorders (Ringstad et al., 2018).

These pathological conditions are associated with a decrease in CSF influx to the glymphatic pathway or reduced clearance efficacy (traumatic brain injury, ischaemic stroke) or both (aging, AD, subarachnoid hemorrhage, idiopathic normal pressure hydrocephalus) (Plog and Nedergaard, 2018; Rasmussen et al., 2018). Nonetheless, it still remains difficult to establish to what extent a primary disruption of the glymphatic system is responsible for the onset of brain pathologies or rather it is a CNS disease that affects this delicate drainage pathway. Even, a mutual detrimental influence between noxious stimuli and interstitial fluid dynamics should be considered.

## Glymphatic System and Periphery: Implications for CNS Disorders

Considering that a healthy human body depends on the correct communication among various integrated systems, it is essential to have a holistic view to better understand and interpret its dynamics under normal and pathological conditions. Then, the classic distinction between the CNS and the body periphery appears now inadequate. In this regard, another important issue to be discussed is the influence of peripheral pathologies on the integrity of CNS paravascular spaces, with possible negative consequences on neuronal activities. For instance, diabetes mellitus impairs glymphatic clearance of interstitial solutes within the hippocampus and hypothalamus of rats, which is correlated with cognitive decline (Jiang et al., 2017).

Again, accumulation of metabolic waste products and noxious substances in the brain ISF may result from liver disease, potentially contributing to neuronal dysfunction and cognitive impairment (Hadjihambi et al., 2019). This was confirmed in a rat model of chronic liver disease obtained through bile duct ligation, where altered glymphatic clearance and reduced AQP4 expression occurs in several brain regions, including the olfactory bulb, prefrontal cortex and hippocampus. These effects are aligned with cognitive/behavioral deficits (Hadjihambi et al., 2019). It has been speculated that, in the advanced phases of liver cirrhosis, glymphatic damage could be the end-stage phenomenon of a cascade of hydrodynamic events. These start from the onset of a vast number of artero-venous shunts in several organs and apparatuses and culminate into a reduction of jugular vein outflow (Gallina et al., 2019). This may in turn induce a reduction of cerebral-venous outflow and consequently, impairment of CSF circulation, derangement of AQP4-based clearance, accumulation of waste molecules and fluids, glymphatic congestion and inflammation.

A variety of general conditions can influence the efficiency of brain clearance from waste products. For instance, not only the level of consciousness, but also body posture (supine, prone, or lateral positions) contributes to glymphatic drainage. An experimental study indicates that the right lateral decubitus, which is natural in rodents at rest, is mostly efficient for glymphatic transport and elimination of waste products, including β-amyloid (Lee et al., 2015). One potential explanation for such an advantage is that the heart is positioned higher, which may favor pumping of blood and greater venous return to increase cardiac stroke volume; in turn, sympathetic tone is reduced, possibly improving glymphatic influx. However, more complex physiological adjustments to different head and body positions (including stretch on the nerves and vessels on the neck) are likely to be involved. Preliminary results were also obtained in patients, where postural changes seem to affect intracranial pressure (Andresen et al., 2016).

As previously mentioned, during sleep, the glymphatic system is highly active in removing waste products. Sleep disturbances are an early correlate of neurodegenerative diseases, including AD and Parkinson’s disease (PD), where they often precede the onset of classic symptoms. In general sleep can be regarded as a neuroprotective factor acting through the glymphatic system (Sundaram et al., 2019). Sleep quality is controlled by circadian rhythms. Recent papers in rodents (Hablitz et al., 2020; Pulido et al., 2020) and Drosophila (Artiushin et al., 2018; Zhang et al., 2018) emphasized the circadian regulation of the glymphatic system, lymphatic drainage and BBB permeability. For instance, in mice, glymphatic CSF influx, and solute clearance from the brain, do vary according to circadian rhythms independent of arousal state (Hablitz et al., 2020). Glymphatic influx and clearance peak during the mid-rest phase of mice, while CSF drainage to the lymph nodes exhibits daily variation opposite to glymphatic influx. This is matched by the perivascular polarization of AQP4, which is highest during the rest phase. An intricate relationship has been documented between neuronal activity and the expression of circadian clock genes within brain ECs, which in turn, orchestrate the activity-dependent control of BBB efflux transport (Pulido et al., 2020).

### Cervical Lymph Nodes and Brain Drainage

The glymphatic pathway is connected to a classic lymphatic network, associated with dural meninges covering the brain, as well as sheaths of cranial nerves and blood vessels, or drains via the olfactory route, then exiting through cranial foramina. This network ultimately drains to deep and superficial cervical lymph nodes, then representing the next step in CNS drainage following the glymphatic system (Ma et al., 2017; Benveniste et al., 2019; Hershenhouse et al., 2019). In rats the uptake of Evans blue tracer from subarachnoid space (cistern magna) was shown to be drained into the meningeal lymphatic vessels and extracranial lymph nodes (Maloveska et al., 2018).

During aging, meningeal lymphatic vessels exhibit decreased vessel diameter and reduced drainage to cervical lymph nodes. Experimental studies in mice showed that ablated or ligated meningeal lymphatics led to an increase in β-amyloid deposition and macrophage recruitment to plaque sites, with a reduced extracellular clearance of altered proteins (Da Mesquita et al., 2018). Behavioral test, including spatial learning and fear memory, deteriorate along with impaired lymphatic function. These data suggest that an impaired efficiency of meningeal lymphatic vessels to drain toward peripheral lymph nodes play a significant role in the pathological accumulation of proteins implicated in neurodegeneration (Hershenhouse et al., 2019). Similar findings were reported for α-synuclein accumulation, a hallmark for a class of degenerative disorders (Zou et al., 2019). The presence in humans of meningeal lymphatic vessels connected with the glymphatic system arouse the hypothesis that clearance of macromolecules implicated in neurodegenerative proteinopathies, such as PD, might also occur through this efflux pathway. Then, an impairment of this drainage might result in α-synuclein accumulation, leading to neurodegeneration (Visanji et al., 2018).

The effects of an impaired drainage of cerebral lymphatic system in the pathogenesis of both ischemic and hemorrhagic stroke was examined. In a model of transient middle cerebral artery occlusion-induced stroke, the blockade of cervical lymphatics worsened cerebral edema and infarct size. Again, an obstruction of meningeal lymphatic vessels after a subarachnoid hemorrhage contributed to the exacerbation of the disease (Sun et al., 2018; Hershenhouse et al., 2019).

The bidirectional connection between the CNS and peripheral immune system through meningeal and cervical lymphatics is also relevant for autoimmunity. In fact, while assisting in the drainage of CSF components, meningeal lymphatics enable immune cells and self-antigen peptides to enter draining lymph nodes (Louveau et al., 2018). This may foster activation of T-cells in periphery while mounting CNS-directed adaptive immune responses (Limanaqi et al., 2019). In fact, peripherally activated T-cells can enter the brain parenchyma by crossing all CNS barriers including the blood-CSF, the blood-leptomeningeal, and the BBB (Shechter et al., 2013; Limanaqi et al., 2019). In line with this, resection of either meningeal lymphatics or deep cervical lymph nodes is beneficial in models of multiple sclerosis (MS), which is characterized by abundant inflammation and infiltration of brain-reactive immune cells throughout the CNS (Phillips et al., 1997; Furtado et al., 2008; van Zwam et al., 2009; Louveau et al., 2018; Hershenhouse et al., 2019).

It is intriguing that besides neuro-immune disorders such as MS, autoimmune mechanisms may also be implicated in classic neurodegenerative disorders such as PD. In fact, nigral dopamine (DA) neurons possess an enhanced sensitivity to the upregulation of major histocompatibility complex I (MHC-I) molecules (Cebrián et al., 2014). Thus, their susceptibility in PD may be related to cytotoxic, CD8 + T-cell-mediated death (Sulzer et al., 2017). This is bound to α-synuclein degradation and subsequent generation of self-antigen peptides for T-cell presentation through neuronal MHC molecules (Cebrián et al., 2014; Sulzer et al., 2017; Ugras et al., 2018). In fact, just like professional antigen presenting cells, DA neurons can internalize, process and load antigens onto MHC-I, especially during pro-inflammatory conditions (Cebrián et al., 2014; Limanaqi et al., 2019). This occurs following either administration of DA precursors, or microglial activation and subsequent cytokines release. In the presence of activated CD8 + T-cells, the cognate antigen/MHC-I complex exposed on the plasma membrane of DA neurons induces T-cells proliferation, and eventually, neuronal death via Fas/Fas ligand and perforin/granzyme pathways. Thus, an apparently paradoxical scenario configures, whereby drainage of α-synuclein to the peripheral lymph nodes may trigger autoimmune attack against brain DA neurons, which remains to be confirmed.

### Glymphatic System and Brain-Gut Axis

Another interesting example of interaction between CNS and periphery is represented by the brain-gut axis. It is now well ascertained that there is a reciprocal communication between brain and gastrointestinal tract. At first, there is a direct transfer of peptides and regulatory proteins across the BBB. Furthermore, gastrointestinal hormones can alter the function of brain ECs, which compose the BBB. Finally, these hormones can affect the secretion from the BBB of substances involved in the regulation of feeding and appetite, such as nitric oxide and cytokines (Banks, 2008).

A large body of evidence shows how gastrointestinal pathologies can affect the CNS bypassing or altering BBB and related pathways, including the glymphatic system. In fact, according to Braak’s hypothesis (Braak et al., 2003) neurodegenerative diseases, in particular PD, may have a peripheral origin. This may occur when putative pathogens enter the mucosa of the gastrointestinal tract, inducing misfolded/aggregated α-synuclein in specific neuron subtypes of the enteric nervous system. These α-synuclein aggregates may finally spread antidromically to the CNS via the vagal preganglionic fibers, up to the dorsal motor nucleus (Natale et al., 2008, 2010, 2011a). In other words, misfolded proteins can propagate via peripheral nervous system (Natale et al., 2011b, 2013; O’Carroll et al., 2020).

**FIGURE 2:**
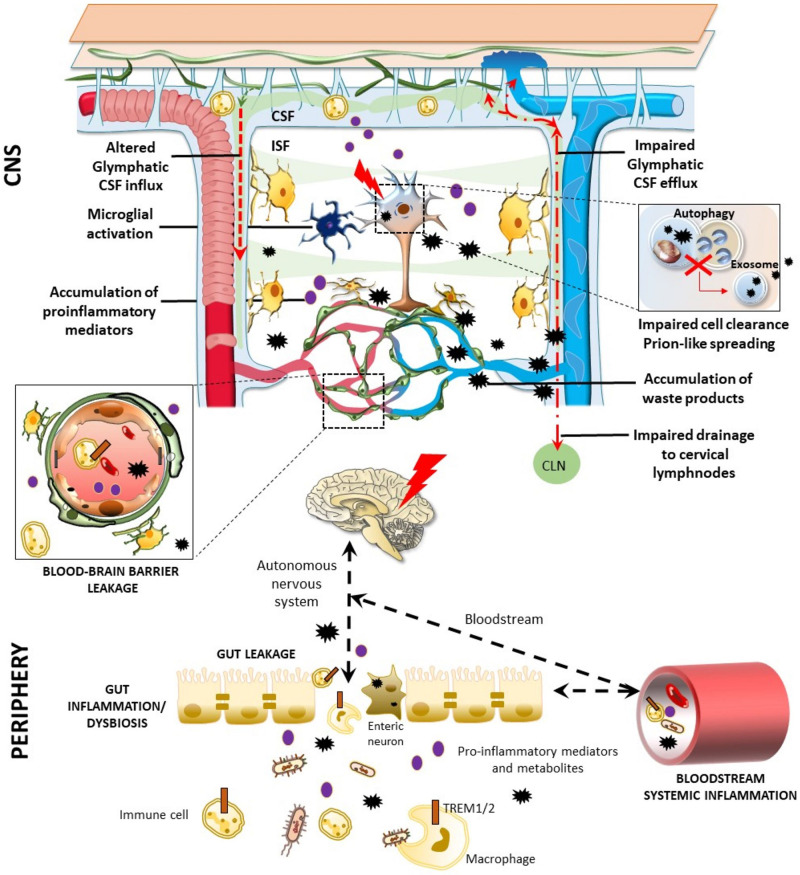
Glymphatic pathway in pathological conditions: a role for the bidirectional gut-brain communication. Alterations of the glymphatic pathway may contribute to the extracellular accumulation of waste products, including altered protein in the brain (black stars). These include alterations in the morphology and drainage capacity of meningeal lymphatic vessels, impairment of CSF influx and efflux, along with the release of several pro-inflammatory cytokines and immune cells. Considering the reciprocal communication which occurs between the brain and the gastrointestinal tract, gut alterations can affect the CNS, and vice-versa. Potentially harmful solutes, including misfolded/aggregated proteins, may spread to the gut through the autonomic nervous system to induce inflammation [brain-first (top-down) type]. In turn, gut dysbiosis, inflammation, and leakage may promote the antidromic spread of potentially harmful molecules to the CNS via the vagal fibers or the bloodstream [body-first (bottom-up) type], bypassing and altering the glymphatic system and the BBB (left insert). These include misfolded/aggregated proteins such as α-synuclein, microorganisms, and also pro-inflammatory cytokines and activated immune cells, such as TREM TREM cells-positive activated macrophages. Extracellular accumulation of waste products related to an altered glymphatic drainage is exacerbated when intracellular clearing systems are impaired (right insert). This is the case of the autophagy pathway, which grants neuronal proteostasis and survival. When autophagy is impaired, extracellular release of undigested, potentially harmful substrates may occur via exosome release.

In spite of these findings, Braak’s hypothesis is still debated. Liddle (2018) collected several data showing that PD may arise in the gut, whereas according to Lionnet et al. (2018) the human autopsy evidence does not seem to support this hypothesis. Finally, it is possible to agree on a compromise when a specific subset of patients affected by PD can be considered within the staging system of Braak (Rietdijk et al., 2017). In particular, two subtypes of PD patients can be recognized: a brain-first (top-down) type, where α-synuclein pathology initially arises in the brain with secondary spreading to the peripheral autonomic nervous system; and a body-first (bottom-up) type, where the pathology originates in the enteric or peripheral autonomic nervous system and then spreads to the brain (Horsager et al., 2020) (Figure 2). Supporting this hypothesis, a novel experimental study showed that α-synuclein fibrils injected into the duodenal and pyloric muscularis layer can spread in the brain, first in the dorsal motor nucleus, and then in the locus coeruleus, and later on, in basolateral amygdala, dorsal raphe nucleus, and substantia nigra pars compacta. Truncal vagotomy and α-synuclein deficiency prevent the gut-to-brain spread of synucleinopathy and associated neurodegeneration and behavioral deficits (Kim et al., 2019).

The role of the gastrointestinal microbiota and their metabolites in modulating brain functions and BBB integrity has rapidly increased over the past years (Cryan et al., 2020; Parker et al., 2020). Interestingly, in a recent work it has been observed that, following fecal microbiota transplant from aged into young mice, a down-regulation of proteins involved in glucose transport across the BBB, such as SlcA1 and A3, takes places, contributing to the dysfunctional bioenergetic system of the aging brain (D’Amato et al., 2020). Furthermore, in an up-to-date review, it was reported that, via the microbiota-gut-brain axis, Triggering Receptors Expressed on Myeloid cells (TREM)-positive activated macrophages along with inflammatory mediators may reach the brain through blood, glymphatic system, circumventricular organs, or the vagus nerve (Figure 2, Natale et al., 2019). This may foster pro-inflammatory reactions in the brain, bridging inflammatory bowel disease and neurological disorders. Similar hypotheses have also emerged on the correlation between gastrointestinal and neurological symptoms of SARS-CoV-2, which may apply indeed to a variety of microorganisms, and also “prionoid” proteins. Once the gastrointestinal tract is invaded, the virus may transit to the CNS through vascular and lymphatic systems, or through the vagus nerve (Bostancıklıoğlu, 2020a,b; Limanaqi et al., 2020). The virus can even infect leukocytes and migrate with them into the brain, or alternatively, viral particles can be directly transported across the BBB to the brain. Again, the virus can invade the peripheral lymphatic vessels which are connected with the glymphatic system, finding a route to the CNS. This suggests that lymph vessels around the gastrointestinal tract, the vascular system itself, or the gut-brain axis via the vagal nerve represent potential peripheral gateways for both pathogen neuroinvasion and prion-like spreading of potentially harmful catabolites to the CNS. If this is the case, accumulation of waste products in the brain would progressively foster pathology due to impairment of (g)lymphatic drainage activity or altered intracellular catabolite scavenge (for example, the autophagy pathway) (Figure 2).

At the same time, perineural spaces surrounding the cranial nerves, including the vagus, are known to provide some level of CSF drainage to peripheral lymphatics (Ma et al., 2017). When considering recent evidence that vagus nerve stimulation enhances CSF tracer influx (Cheng et al., 2020), the top-down hypothesis of neurodegeneration seems to take over. Although a correlation between glymphatic clearance of misfolded proteins and the vagus nerve remains to be investigated, some insights can be provided by the recently described ocular glymphatic system. Following up experimental data that documented retrograde CSF inflow to the paravascular spaces in the optic nerve, it was demonstrated that an eye-to-CSF pathway supports clearance of waste products from the retina and vitreous (Wang et al., 2020). This occurs in opposite direction as compared to CSF drainage, and neural activity seems to play a role on the rate of fluid fluxes, as light stimulation promotes fluid drainage and β-amyloid clearance. After traversing the lamina barrier through an ocular-cranial pressure difference mechanism, intra-axonal Aβ is cleared via the paravenous space and subsequently drained to lymphatic vessels. Apart from providing a potential link between neurodegenerative and ocular diseases, these findings open novel avenues for further experimental studies aimed at dissecting the role of the glymphatic system as a kernel connecting CNS and periphery.

## Author Contributions

GN drafted and wrote the manuscript. FL, CB, FM, FN, and SP-A contributed to the literature review, manuscript writing, and editing. FL made art-work. FF coordinator the manuscript, he critically revised the manuscript for important intellectual content. All authors contributed to the article and approved the submitted version.

## Conflict of Interest

The authors declare that the research was conducted in the absence of any commercial or financial relationships that could be construed as a potential conflict of interest.
